# Physician perspectives on the burden and management of asthma in six countries: The Global Asthma Physician Survey (GAPS)

**DOI:** 10.1186/s12890-017-0492-5

**Published:** 2017-11-23

**Authors:** Kenneth R. Chapman, David Hinds, Peter Piazza, Chantal Raherison, Michael Gibbs, Timm Greulich, Kenneth Gaalswyk, Jiangtao Lin, Mitsuru Adachi, Kourtney J. Davis

**Affiliations:** 1 University of Toronto, Toronto, Canada; 2 Real World Evidence & Epidemiology, GlaxoSmithKline, 1250 South Collegeville Road, Collegeville, PA 19426 USA; 3 Five Dock Family Medical Practice, Five Dock, Australia; 4 Bordeaux University, Bordeaux, France; 5 Global Respiratory Franchise, GlaxoSmithKline, Brentford, UK; 6 University Medical Centre Giessen and Marburg, Philipps-University Marburg, Marburg, Germany; 7 Abt SRBI, Silver Spring, MD USA; 8 China-Japan Friendship Hospital, Beijing, China; 9 International University of Health and Welfare, Tokyo, Japan

**Keywords:** Asthma, surveys and questionnaires, guideline adherence, patient compliance, physicians’ practice patterns, disease management

## Abstract

**Background:**

Despite recognition of asthma as a growing global issue and development of global guidelines, asthma treatment practices vary between countries. Several studies have reported patients’ perspectives on asthma control. This study presents physicians’ perspectives and strategies for asthma management.

**Methods:**

Physicians seeing ≥4 adult patients with asthma per month in Australia, Canada, China, France, Germany, and Japan were surveyed (*N*=1809; ≈300 per country). A standardised questionnaire was developed for this study and administered by telephone, online or face-to-face. Statistics were weighted to account for the sampling scheme.

**Results:**

Physicians estimated that 71% of their adult patients received maintenance medication, with adherence monitored by 76–97% of physicians. Perceived major barriers to patient adherence included: patients taking treatment as needed; acceptance of symptoms; and patients not perceiving treatment benefits. Written action plans (37%) and technology (15%) were seldom employed by physicians to aid patients’ asthma management. Physicians rarely (10%) used validated patient-reported questionnaires to monitor asthma control, instead monitoring selected symptoms, exacerbations, and/or lung function measurements. Awareness of single maintenance and reliever therapy (SMART/MART) varied among countries (56–100%); although most physicians (72%) had prescribed SMART/MART, the majority (91%) co-prescribed a short-acting bronchodilator at least some of the time.

**Conclusions:**

These results show that physicians generally do not employ standardised tools to monitor asthma control or to manage its treatment and that despite high awareness of SMART/MART, the strategy appears to be commonly misapplied. Better education for patients and physicians is required to improve asthma management and resulting patient outcomes.

**Electronic supplementary material:**

The online version of this article (10.1186/s12890-017-0492-5) contains supplementary material, which is available to authorized users.

## Background

Asthma is a global health problem that imposes a substantial burden on healthcare systems and on society through impaired quality of life and loss of productivity in the workplace [1, 2]. Despite significant research effort into the development of effective therapies and evidence-based treatment guidelines [3–5], individuals with asthma throughout the world do not achieve asthma control [6–10].

Both global and national guidelines have been developed to standardise and improve asthma management, thereby aiming to help patients optimise asthma control. After a collaboration between the American National Heart, Lung and Blood Institute and the World Health Organization in 1993, the Global Initiative for Asthma (GINA) was established and its ‘Global Strategy for Asthma Management and Prevention’ reports have been updated annually since 2002 [11]. Medical associations in many countries have also produced national guidelines for the management of asthma [12–14]. These guidelines provide both therapeutic and non-pharmacological recommendations for optimal management of asthma, as well as defining levels of asthma control.

Guidelines have evolved with changing treatment patterns in asthma, including the establishment of inhaled corticosteroids (ICS) as the cornerstone of treatment for patients with persistent asthma, and the incorporation of ICS combined with long-acting β_2_-agonists (LABAs) [11]. Guidelines have also evolved with changing dosing strategies such as the option of prescribing an ICS/formoterol combination in a single inhaler as both maintenance and reliever therapy, rather than using separate formoterol or another short-acting β_2_-agonist (SABA) for rapid symptom relief (rescue use) [11]. Use of single maintenance and reliever therapy (known as SMART or MART – for simplicity MART will be used hereafter) has been reported to show similar efficacy in clinical trial settings compared with alternative ICS/LABA combinations with additional SABA use, especially in patients with a history of exacerbations [3, 15].

Multiple studies of patients with asthma have revealed the variable levels of asthma control achieved worldwide. The Asthma Insights and Reality (AIR) studies of patients from regions including North America [16], Europe [6], and the Asia-Pacific region [7] in the late 1990s and early 2000s demonstrated that levels of asthma control were substantially below the goals set out in the GINA guidelines [11] and that patients tended to underestimate the severity of their asthma symptoms [6, 7]. More recently, the Asthma Insight and Management (AIM) surveys of patients in the USA [17], Latin America [8], Canada, and Europe [18], and the REcognise Asthma and LInk to Symptoms and Experience (REALISE) surveys of European [9] and Asian [10] patients found that the issues identified in the AIR studies are still present.

Several single-country studies have also highlighted the variability of asthma control: one survey of patients in China reported that only 28.7% achieved total control as defined in the GINA guidelines [19], while a second survey reported this figure to be 40.5% [20]. A Canadian study of both patients and primary care physicians designed to eliminate any potential bias associated with telephone-based surveys of asthma control also reported low levels of total asthma control (23%), and found that physicians overestimated patients’ levels of control [21].

While most surveys to date have examined the perspectives of patients, it is also important to understand the perspectives of physicians treating these patients. The 2005 Global Asthma Physician and Patient (GAPP) study in 16 countries reported differences between ‘real-world’ treatment practices and those recommended in the GINA guidelines [22]; these have been similarly observed in several other country-specific surveys [23–25]. The GAPP survey also revealed considerable differences between patients’ and physicians’ perspectives on asthma education, treatment adherence and potential side effects of medication [22]. The second phase of the REALISE Asia study surveyed 375 physicians across Asia and reported differences between physicians’ and patients’ definitions of asthma control, with both groups overestimating patients’ levels of control [26]. These studies demonstrate the importance of surveying physicians as well as patients to gain a full understanding of perspectives and management practices.

The present study, the Global Asthma Physician Survey (GAPS), aimed to provide a current picture of asthma management practices in different countries by assessing physicians’ perspectives regarding the burden, management, and treatment of asthma, including management with and ‘real-world’ implementation of MART dosing.

## Methods

### Study Design and Participants

GAPS (GSK study number PRJ2509) was a cross-sectional survey of general practice or internal medicine physicians in Australia, Canada, China, France, Germany, and Japan who routinely treated adult patients with asthma (≥4 patients per month) conducted between May and September 2015. In France and Germany, participants were drawn from a probability-based national sample frame proportional to the distribution of physicians by geographic strata. In Australia and Japan, participants were sampled from a nationally representative online opt-in panel. In Canada, participants were drawn from both a national sample frame and a nationally representative opt-in panel, while in China, the sampling plan was consistent with the tiered system of hospitals that deliver primary care: internal medicine physicians were recruited from hospitals in five major cities (Beijing, Shanghai, Guangzhou, Chengdu and Wuhan; approximately 60 physicians per city), with the proportion of physicians from each hospital within a city adjusted according to the size (tier) of the hospital.

### Survey Administration

All surveys were conducted in the local language(s). In France and Germany, surveys were administered by telephone. In Australia and Japan, surveys were administered online. In Canada, surveys were administered both by telephone (103 physicians, national sample frame) and online (204 physicians, opt-in panel). In China, participants were interviewed by professional interviewers and offered either a face-to-face interview with paper questionnaires or a telephone interview.

### Questionnaire

The questionnaire was developed in conjunction with key external experts (the authors) in the relevant countries with knowledge of local asthma treatment and management practices. It was designed to assess: physicians’ beliefs about asthma; knowledge of and adherence to asthma guidelines; perceived barriers to patients’ adherence; and asthma management and treatment practices, including management with MART. The full questionnaire (in English; translations were employed as necessary and reviewed by national experts) is provided in the Additional file 1, along with the relevant national guidelines relating to MART in each of the six countries studied (Additional file 2: Table S2).

### Data Analysis

Physicians’ responses to survey questions were tabulated and described. Overall statistics were weighted by the number of physicians in each country to adjust for variations in country size. Country-specific data were weighted by known demographic parameters of that country from the sampling frame and available government statistics using the software SAS version 9.3 (Cary, NC, USA).

## Results

Overall 1809 physicians from Australia, Canada, China, France, Germany, and Japan completed the survey, with approximately 300 physicians from each country (Table 1). Physician age, gender and practice type varied considerably between countries. Physicians in France, Germany, and Japan tended to be older, while physicians in France and Japan were less likely to have ever participated in continuing medical education on asthma.

**Table 1:** Demographics of physician survey respondents

	Total *N*=1809	Canada *N*=307	France *N*=301	Germany *N*=300	Australia *N*=300	China *N*=300	Japan *N*=301
Age (years)							
<35	7	13	10	1	9	12	-
35–44	25	25	16	15	23	46	13
45–54	30	27	25	30	31	35	29
55–64	28	23	40	39	25	8	41
>65	9	12	9	16	13	-	16
Gender							
Male	61	56	63	59	59	40	86
Type of practice							
Single specialty or solo GP	32	56	62	85	12	-	23
Multi-speciality or group GP	36	21	36	14	88	5	72
Hospital or hospital clinic	31	15	1	-	-	95	5
Continuing medical education on Asthma (ever)	79	94	69	98	92	92	54
Mean number of patients with asthma seen per month (n)	40	56	15	31	68	37	51
Ages of patients seen^a^							
Paediatric	13	21	24	5	33	2	11
Adolescent	12	17	19	9	21	6	11
Adult	78	63	57	86	46	94	80
Mean percentage of adult patients on maintenance medication	71	73	63	77	66	68	77

Values are % except where stated otherwise. ‘-’ represents values of 0% or <1%. Percentage values within categories may sum to <100% or >100% due to rounding and weightings applied. GP, general practitioner/physician. ^a^Paediatric: <12 years; adolescent: 12–17 years (12–15 years in Canada); adult: ≥18 years (≥16 years in Canada)

Response rates ranged from 12% in Canada to 54% in China, and the average survey time was 21 minutes. Physicians’ estimates of the proportions of their adult patients treated with maintenance asthma medication were relatively consistent (71% overall), ranging from 63% (France) to 77% (Germany and Japan).

### General Perspectives on Asthma

Overall, 83% of physicians (range: 69% in France to 88% in Australia and Germany) surveyed believed that the long-term health outlook for patients with asthma has improved compared with 10 years ago. The primary reason for this in all countries, given by 90% of these physicians, was the increased options for and/or improvement in asthma medications and devices. There was considerable variation between countries in other reasons given for the perceived improved outlook over the last 10 years (Additional file 2: Table S1): improved understanding by physicians was selected by only 2% of respondents in Japan, but by 45% of respondents in China, while improved understanding by patients varied from <1% (Australia and Japan) to 38% (China).

The major barriers to medication adherence were perceived to be: i) patients only taking maintenance treatment as needed; ii) patients adapting to or tolerating ongoing asthma symptoms; and iii) patients not perceiving the benefit of the treatment. Differences in views between countries are presented in Table 2.

**Table 2:** Percentage of physicians reporting each barrier to patient adherence as a major problem

% of physicians reporting barrier as a major problem	Total *N*=1809	Canada *N*=307	France *N*=301	Germany *N*=300	Australia *N*=300	China *N*=300	Japan *N*=301
Patients only take treatment when needed	61	72	66	31	71	57	68
Patients believe asthma symptoms are normal	58	41	57	30	42	61	78
Patients don’t perceive the benefit of the treatment	56	40	55	41	43	45	81
Poor inhaler technique	53	52	61	43	52	27	81
Low patient education level or poor understanding of disease	52	52	60	54	54	31	70
Low level of patient engagement in disease	52	41	63	39	47	31	80
Forgetfulness	45	39	54	24	43	20	78
Troublesome side effects	45	7	18	15	12	63	72
Corticosteroids are perceived as harmful	43	30	20	40	31	47	61
The cost of medications	37	56	6	16	26	42	55
Inconvenience of dosing schedule	33	19	31	15	22	20	62

### Perspectives on Asthma Management

Of physicians surveyed, 87% reported monitoring adherence to maintenance medication (range: 76% in Canada to 97% in China), while monitoring reliever medication usage was reported by 89% of physicians. Most physicians reported monitoring adherence and reliever medication use through patient interactions: asking directly (91% and 93% of physicians overall for maintenance and reliever use, respectively) or estimating from patient interviews (80% and 78%, respectively). Fewer physicians estimated medication use by objective means such as the timing of prescription refill requests (57% and 50% for maintenance and reliever medications, respectively) or checking prescription labels for the date dispensed (38% and 28% for maintenance and reliever medications, respectively). Trends were consistent between countries.

Written action plans were infrequently implemented with patients. Physicians estimated that only 37% of their patients were issued with a written asthma action plan, though this varied by country from 30% (China) to 50% (Japan). Technology was generally not used in asthma management: overall only 15% of physicians reported using any mobile, online or digital tools, ranging from 8% (Japan) to 25% (China).

Physician practices for assessing asthma control also varied considerably among countries (Table 3); in particular, the data from physicians in China differed noticeably from other countries. Monitoring symptom frequency was the most commonly used method in China, used by 95% of physicians, but was used by only 8% of physicians in Japan, where monitoring exacerbations was the most frequent response. In France and Germany, lung function measures (spirometry) were the most frequently used method to assess control. Monitoring use of reliever SABAs for symptom control was the most common method of assessing control in Australia and Canada. Use of validated patient-reported questionnaires was low in all countries (used by 10% of physicians overall).

**Table 3:** Practices for assessing asthma control

% of physicians using this method	Total *N*=1809	Canada *N*=307	France *N*=301	Germany *N*=300	Australia *N*=300	China *N*=300	Japan *N*=301
Lung function measurement with spirometry (e.g. FEV_1_ or peak flow)	42	28	46	57	47	54	24
Frequency of symptoms	41	15	28	31	12	95	8
Frequency of exacerbations	41	41	31	9	37	59	43
Frequency of night-time awakenings	30	31	8	6	33	70	8
Interference with normal activities (excluding work)	27	21	15	3	16	60	14
Use of reliever SABAs for symptom control	23	42	17	5	53	37	5
Interference with work or household work	20	16	4	2	7	58	1
Validated patient-reported outcomes (ACT, ACQ)	10	5	29	8	2	13	1
Symptoms (unspecified)	7	17	1	3	32	1	12
Medication use and frequency	6	15	1	6	15	-	10
Patient or family feedback	6	12	1	12	15	-	9
Ability to exercise/exertion	5	15	-	2	19	-	8
Induced sputum measurement	5	1	1	-	2	15	-
Other responses combined	37	54	33	45	68	2	61

Responses to survey question: How do you assess asthma control in your adult asthma patients?

Only answers with a response rate of ≥15% in at least one country have been included. ‘-’ represents values of 0% or <1%

*ACQ* Asthma Control Questionnaire, *ACT* Asthma Control Test, *FEV*_*1*_ forced expiratory volume in 1 second, *SABA* short-acting β_2_-agonist

The majority of physicians surveyed believed that their adult patients received the treatment that the physician considered to be best for them at least most of the time (86% overall, ranging from 76% in Japan to 97% in Germany). The most commonly selected reason (given by 59% of physicians overall) why patients do not receive the treatment that physicians believed to be the best for them was that patients failed to understand the importance of using the medication. The next most commonly given reason (37% overall) was ‘patient factors’ such as compliance, lifestyle/habits and poor follow-up; this reason was rated very differently between countries, given by 84% of physicians in Australia and 63% in Canada, but only 2% in China.

### Perspectives on MART

**Fig. 1:**
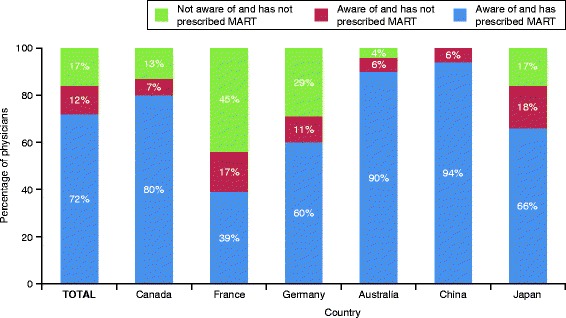
Awareness and prescribing of MART in different countries. MART, maintenance and reliever therapy. Percentage values within categories may sum to <100% or >100% due to rounding and weightings applied; calculations were performed using weighted *n* values

The majority of physicians surveyed (n=1479, 83%) reported being aware of MART; of these physicians, the majority (n=1286, 86%) had prescribed MART. Awareness and use of this strategy varied considerably by country (Fig. 1). Subsequent questions regarding MART administration were asked only of physicians who answered affirmatively that they had ever prescribed MART.

Physicians’ sources for information about MART were mainly scientific conferences (54% overall; from 17% in France to 75% in China) and pharmaceutical sales representatives (49% overall; from 40% in China to 74% in Australia) rather than from local (31%) or GINA (30%) guidelines. Conversely, many physicians in China reported obtaining their information from local (77%) or GINA (77%) guidelines. The proportion of ICS/LABA fixed-dose combination inhalers prescribed as MART was 42% overall, varying from 33% (Germany) to 47% (China).

Physicians’ perspectives on MART are summarised in Table 4. The most common reason for prescribing MART overall was the severity of symptoms (cited by 35% of physicians); however, patient factors (such as age, understanding, and compliance) were the most common reason in Australia, Canada, and Germany. In Japan, both patient factors and the presence or risk of exacerbations were the most common reasons to prescribe MART. The mild nature of symptoms was the most common reason overall (38% of those reporting situations where MART would not be prescribed) for not prescribing MART.

**Table 4:** Physicians’ perspectives on MART

% of physicians giving response	Total *n*=1286	Canada *n*=248	France *n*=112	Germany *n*=180	Australia *n*=269	China *n*=281	Japan *n*=196
Reasons for prescribing MART							
Patients with severe symptoms (daytime/night-time symptoms ≥4 days/week)	35	3	15	11	1	79	3
Patients with moderate symptoms (daytime/night-time symptoms 2–3 days/week)	33	5	16	10	1	72	6
Patients with/at risk of exacerbations	30	7	12	5	9	51	29
Greater convenience for the patient as only one inhaler is required	22	27	32	15	28	29	6
Patient factors (knowledge/understanding, compliance, age)	20	54	5	17	65	-	29
Reasons for not prescribing MART^a^							
Patients with more mild symptoms	38	10	8	4	13	64	6
Patients with poor adherence	25	13	34	18	18	29	18
Patients with severe symptoms (daytime/night-time symptoms ≥4 days/week)	19	14	14	17	18	25	5
Patients with lots of co-morbidities	14	18	3	16	16	15	10
Patient factors (knowledge/understanding, compliance, age)	10	30	14	10	37	-	21
Prescription of SABA/bronchodilator with MART							
Always	19	13	27	10	6	33	3
Most of the time	30	19	29	29	23	42	18
Some of the time	42	55	37	52	50	23	63
Never	8	13	7	9	21	1	13
Reasons for prescribing SABAs / bronchodilators with MART^b^							
Patients want an extra reliever to feel safer	35	14	28	36	14	59	8
Patient familiarity with short-acting-beta medicine	32	19	13	13	18	59	8
Patient convenience – having an extra reliever on hand	27	4	13	5	3	56	2
Patients are accustomed to having an extra reliever	20	5	19	6	6	35	8
MART dosing instructions provided to pharmacy							
Don’t write anything different to normal dosing	32	25	10	29	23	38	37
Write Maintenance Plus Reliever language as a standard choice	26	19	27	8	18	41	15
Have to create my own text noting maintenance frequency and PRN	21	35	54	33	36	1	24
Write ICS/LABA as both maintenance and reliever	15	18	7	13	20	16	17
MART dosing instructions provided to patients							
Verbal instructions to use the ICS/LABA as relief as well as maintenance	87	89	91	83	73	93	82
Written instructions to use the ICS/LABA as relief as well as maintenance	59	45	84	81	66	71	24
Verbal instructions to use the ICS/LABA as relief but not maintenance	14	7	36	20	6	16	6
Written instructions to use the ICS/LABA as relief but not maintenance	6	4	30	18	3	1	1
Time/effort required for MART prescribing vs. other maintenance medications^c^							
Much more time and effort	14	4	1	6	5	24	11
Somewhat more time and effort	40	32	21	38	40	45	42
About the same	29	38	68	43	29	19	20
Somewhat less time and effort	13	19	9	9	18	7	21
Much less time and effort	5	7	0	4	7	5	6

Questions regarding MART administration were asked only of physicians who had prescribed MART. ‘-’ represents values of 0% or <1%. For questions with more than five possible responses, the five reasons with the highest overall response rates (with overall response rates of ≥10%) have been included. Percentage values within categories may sum to <100% or >100% due to rounding and weightings applied

*ICS* inhaled corticosteroid, *LABA* long-acting β_2_-agonist, *MART* maintenance and reliever therapy, *PRN* pro re nata (as needed), *SABA* short-acting β_2_-agonist

^a^Among physicians responding yes to there being situations where MART would not be prescribed: Total (*n*=805), Canada (*n*=151), France (*n*=70), Germany (*n*=93), Australia (*n*=157), China (*n*=259), Japan (*n*=75); ^b^Among physicians reporting prescribing SABA with MART some of the time, most of the time, or always: Total (*n*=1127), Canada (*n*=211), France (*n*=104), Germany (*n*=165), Australia (*n*=209), China (*n*=277), Japan (*n*=161); ^c^Excluding physicians responding ‘Don’t know’ or ‘Refuse’: Total (*n*=1283), Canada (*n*=248), France (*n*=112), Germany (*n*=179), Australia (*n*=269), China (*n*=281), Japan (*n*=194)

**Fig. 2:**
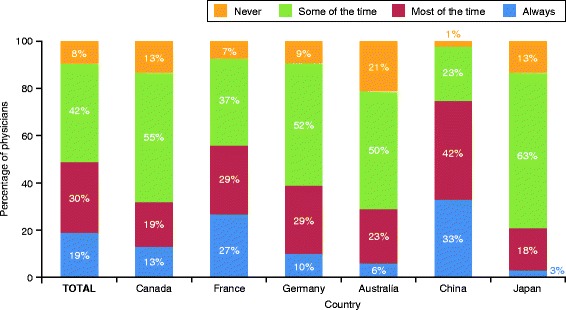
Prescription of SABAs or bronchodilators in addition to MART. MART, maintenance and reliever therapy; SABA, short-acting β_2_-agonist. Percentage values within categories may sum to <100% or >100% due to rounding and weightings applied

The majority of physicians (91% overall) prescribed a SABA or bronchodilator in addition to MART at least some of the time (Fig. 2). A substantial proportion of physicians prescribed additional bronchodilators either most of the time or always (49% overall, ranging from 21% in Japan to 75% in China), while a similar proportion prescribed additional bronchodilators some of the time (42% overall, ranging from 23% in China to 63% in Japan). The primary reason overall (cited by 35% of physicians) for prescribing additional reliever medication was that it made patients feel safer; patient familiarity with SABA medicine and patient convenience were also commonly selected (Table 4).

Overall, 32% of physicians did not provide any specific instructions to pharmacies when prescribing MART. Instructions to patients to use the ICS/formoterol combination as relief as well as maintenance were mainly verbal (87% overall) and/or written (59% overall, varying considerably between countries from 24% in Japan to 84% in France). When asked about the time and effort required for MART prescribing compared with other maintenance medications, the majority of physicians reported that ‘somewhat more’ (40% overall, ranging from 21% in France to 45% in China) or ‘about the same’ (29% overall, ranging from 19% in China to 68% in France) time and effort was required. However, 13% of physicians reported that ‘somewhat less’ time and effort was required (range: 7% in China to 21% in Japan) and only 5% (range: 0% in France to 7% in Canada and Australia) reported that ‘much less’ time and effort was required. The proportion of physicians reporting that ‘much more’ time and effort was required was low (14%), ranging from 1% in France to 24% in China. The majority of physicians believed that their patients understand MART ‘somewhat well’ (60%) or ‘very well’ (36%).

## Discussion

Despite variations in asthma management practices among countries, several overall trends were observed. The majority of physicians agreed that the outlook for asthma patients has improved in the last 10 years (mainly through the availability of better and more devices and medications). It is somewhat surprising that physicians did not rate other reasons more highly, such as better understanding of the disease by physicians and patients, education/awareness, adherence to treatment regimens and better engagement of patients in managing their disease. This is despite the clear directions in treatment guidelines for many years that these issues are important in improving asthma care [11]. There was widespread concern about patient adherence, with the main barriers to adherence focusing on patients’ lack of understanding of the disease and treatments. Patients’ lack of understanding was also the most commonly cited reason for patients not receiving treatment that physicians believed was best for them, while institutional factors (such as cost or insurance coverage) were cited much less frequently. Awareness of MART was high, yet few physicians used this strategy as recommended in the GINA guidelines [11] and as per licence, instead prescribing additional SABAs or bronchodilators.

Considerable variation among countries was observed in preferred methods for assessing asthma control. There was a noticeable discrepancy between the measurements recommended by GINA (frequency of daytime symptoms, night-time awakenings, reliever medication use and extent of activity limitation) [11] and those used in several countries. In particular, physicians in Germany had low use of these GINA measurements, instead mainly using lung function measurements (recommended by GINA for assessing the risk of poor asthma outcomes rather than the level of asthma control [11]) reflecting the advice given in German national guidelines [27, 28]. Physicians in China had relatively high use of the GINA criteria; notably 60% of physicians used activity limitation to assess control, while in other countries this measure was used by ≤21% of physicians. The reported use of standardised, validated patient-reported outcomes (such as the Asthma Control Test [ACT]) was consistently low, particularly in Australia, Canada, China, Germany, and Japan, despite their confirmed reliability [29, 30]. These findings suggest that the barriers to the integration of guidelines into routine clinical practice need to be addressed through well-designed interventions with both patients and healthcare providers.

GINA guidelines recommend the use of written action plans for all patients [11]; however, their use was found to be relatively low in this study (30–50%). In addition, the use of written plans reported was higher than the use of patient-reported outcomes for assessing asthma control, demonstrating that these tools are generally not used together, as advocated by GINA. Technological advances may aid the use of written action plans; however, despite the increasing use of technology in healthcare, its use in asthma management in this study was extremely low, perhaps due to the lack of availability or physician recommendation of suitable digital tools, or a lack of interoperability between digital tools on patient devices and electronic medical record systems. The increasing and reportedly successful use of technology in diabetes management [31] sets an example that might inspire further research into the development and everyday use of suitable digital tools for use in managing asthma.

The overall trends found across the countries can mask some interesting trends found at a national level. This is most evident in China, where the sample population differed from the other countries in terms of physicians’ age and gender distributions, and working environment (primary care asthma management is provided by internal medicine physicians in hospitals). Responses of physicians in China often differed from those of physicians in the other countries; in some cases, the considerable differences skewed the overall data. Physicians in China had the highest awareness and use of MART, and unlike in other countries, physicians reported obtaining information about MART from local and national guidelines. Despite this awareness, they also had the highest prevalence of prescribing additional SABAs or bronchodilators with MART, suggesting a possible lack of understanding of the treatment, lack of belief in its effectiveness, or demand from patients for the ‘safety net’ of having a separate reliever.

Previous surveys of patients with asthma (notably AIR [6, 7, 16], AIM [8, 17, 18, 32] and REALISE [9, 10]) have emphasised patients’ lack of both understanding of treatment and belief in its effectiveness when used in accordance with guidelines. Patients also communicated low expectations of achievable control and a high tolerance of symptoms, as demonstrated by the discrepancy between patients’ assessments of their asthma control and the extent of their control according to guideline criteria [6–10]. This is despite the demonstration that guideline-defined control is achievable in most patients [33]. The perceived barriers to patient adherence and reasons for patients not receiving the treatment physicians considered best for them reported in this study are consistent with these previous findings, suggesting that most physicians are at least aware of these issues. Some of the perceived barriers to adherence relate to health literacy, emphasising the need to consider this in patient education practices [34].

Another important issue revealed in previous surveys of both patients [32] and physicians [22] is the discrepancy between treatment and management practices recommended in guidelines and those actually experienced by patients. This is due to both a difference in physicians’ practices from those advised and a lack of compliance in patients. In terms of treatment, previous surveys have examined use of ICS-containing regimens but not specific use of MART; however, our findings in relation to MART use are consistent with the lack of adherence to guidelines emphasised previously. In terms of management, the low use of written management plans reported in this study, despite their recommendation in guidelines, is consistent with previous findings [32]. This may reflect the debate about the extent of the benefits of written management plans [35] since these benefits were first presented in 2004 [36]. The proportion of physicians employing written plans in Germany recorded in our study (37%) is lower than published data from the German Disease Management Programme (DMP) evaluation would suggest [37]. Another study reported this discrepancy in use of written plans between German patients enrolled in the DMP and those not enrolled [38], demonstrating both the need to assess a representative population when calculating overall population estimates and the potential benefit of enrollment on such a structured programme. The low use of patient questionnaires for assessing asthma control found in this study is also consistent with previous findings [32], yet the overall or country-specific reasons explaining this remain unclear.

This study has provided data of over 1800 physicians’ perspectives over four continents in 2015; however, it is limited to countries with higher levels of healthcare resources and, therefore, cannot be generalised to all countries. The physicians included in the sampling frame were generalists; it is possible that the perspectives and behaviours of specialists might differ (for example, a recent study comparing asthma management in different types of physicians in Italy found that use of the ACT was considerably higher in pneumologists and allergologists than in general practitioners [39]). However, the perspectives of generalists are of high importance since patients are most commonly assessed by a general practitioner, at least as a first-line approach. As for all surveys, the results are subject to inaccuracy in physicians’ reporting or recall, which would lead to a non-differential bias. A further limitation is the difficulty in capturing fully the impact of the different healthcare systems and the different types of samples in the study results, despite adjustment for sampling weights. The sampling schemes used may have affected the response rates in different countries, and the differences in healthcare systems were likely to have contributed to the observed variations in practice type. Similarly, cultural and study administration differences cannot be excluded as explanations for some of the observed variation between countries. This study presents only the views of physicians; the addition of patients’ opinions would have provided more insight, for example, into the difference between patients’ understanding as perceived by physicians, and their own reported understanding.

This study primarily revealed the widespread need for improved physician and patient education about asthma. Patient outcomes have been shown to benefit from physicians’ continued medical education in asthma [40, 41] thus increased education for primary care physicians should be prioritised, particularly in regards to the practices advised in current global and local guidelines for asthma management. This education should focus on the perceived barriers to improved adherence (e.g. educating patients on proper use of medication, identifying asthma symptoms, and inhaler technique), use of written management plans and validated patient questionnaires for assessing asthma control, and on the prescription of medication as indicated in guidelines. Physicians’ responses to several questions highlighted their tendency to rely upon advances in therapeutic intervention to improve the quality of asthma care, rather than the non-therapeutic aspects of asthma management (such as the use of written management plans and validated patient-reported outcomes). Future research might be advisable into the time that would be required in a patient consultation to implement guideline recommendations properly, in comparison with the actual time available to primary care physicians for patient consultations.

Effects of physicians’ ages and genders on their prescribing practices are perhaps under-investigated; most studies have focused on patient demographics. However, the complex nature of the physician–patient relationship (one study found that patient demographics affected physicians’ adherence to guidelines [42]) provides an interesting area for further research. Research into this relationship to date has enabled the development of frameworks for shared decision-making, a collaborative process between physicians and patients aiming to facilitate joint decisions about patients’ healthcare management where there are multiple treatment options [43].

Another potential area for future research is the use of electronic devices for monitoring symptoms, health status and medication use which easily integrate with electronic health records and/or pharmacy dispensing data. Digital solutions may enable easier and more accurate monitoring of patients’ adherence and asthma control, allowing for early and effective interventions to reduce risks and improve outcomes [44].

## Conclusions

In conclusion, while asthma management practices vary by country, these data suggest that better education for both patients and physicians is required globally, particularly in the use of asthma management plans combined with a much wider use of standardised, validated asthma management tools. Consideration of physicians’ perspectives and local challenges, such as varying healthcare systems and cultural preferences, in removing the barriers to allow physicians to deliver guideline-recommended care should ultimately improve asthma outcomes.

## Additional files

Additional file 1: Questionnaire used in the GAPS survey. (PDF 809 kb)

Additional file 2: **Table S1.** Reasons given for the perceived improved outlook for asthma patients in the last 10 years. **Table S2.** Products approved for maintenance and reliever treatment of asthma in the countries studied. (PDF 202 kb)

## Abbreviations

ACT: Asthma Control Test
AIM: Asthma Insight and Management
AIR: Asthma Insights and Reality
DMP: Disease Management Programme
GAPP: Global Asthma Physician and Patient
GAPS: Global Asthma Physician Survey
GINA: Global Initiative for Asthma
ICS: inhaled corticosteroids
LABAs: long-acting β_2_-agonists
MART: maintenance and reliever therapy
REALISE: REcognise Asthma and LInk to Symptoms and Experience
SABA: short-acting β_2_-agonist
SMART: single maintenance and reliever therapy
