# Supplementary material for: Physician perspectives on the burden and management of asthma in six countries: The Global Asthma Physician Survey (GAPS)
Source: BMC Pulm Med. 2017 Nov 23;17:153. doi: 10.1186/s12890-017-0492-5 (PMC5701503; doi:10.1186/s12890-017-0492-5)
Supplement: Supplementary file 1 — Questionnaire used in the GAPS survey. (PDF 809 kb) [file 12890_2017_492_MOESM1_ESM.pdf]

Physician Perspectives on the Burden and Management of Asthma in Six Countries: the Global Asthma Physician Survey (GAPS)

Supplementary Information File 2: Questionnaire used in the GAPS survey

The following questionnaire was used (translated into local languages in conjunction with local experts where necessary) in this study.*

Q1. In an average month, approximately how many adult asthma patients do you see in an
[IF COUNTRY is Canada, France, Germany: ambulatory care clinic**]**
[IF COUNTRY is Australia: general or family practice**]**
[IF COUNTRY is Japan: OUTPATIENT clinic], that is, patients not admitted to the hospital for overnight stays?
[**IF China:** In an average month, approximately how many adult asthma patients do you see, excluding patients who are admitted to the hospital for overnight stays?]

[**INTERVIEWER INSTRUCTION:** Confirm after data entry**]**.

Number: _________ [RANGE 0-200]
IF LESS THAN 4 ➔ SCREEN OUT

998 (VOL) Don't know ➔ **SCREEN OUT**
999 (VOL) Refused ➔ **SCREEN OUT**

SCREENOUT: I'm sorry but you are not eligible for this survey. Thank you for your time.

Q3. We would like to understand the age of asthma patients you see in terms of the percentage that are pediatric, adolescent, and adult. Approximately what percent of your asthma patients are…?
[**INTERVIEWER INSTRUCTION:** Confirm after data entry**]**.

a. Less than age 12
b. Age 12 to 17 [**IF COUNTRY is Canada**: Age 12 to 16]
c. Age 18 and older [**IF COUNTRY is Canada**: Age 16 or older]

Number: _________ [RANGE 0-100]
[DATA VALIDATION: Percentages must sum to 100]

998 (VOL) Don't know
999 (VOL) Refused

Q5. Compared to 10 years ago, would you say the **LONG-TERM HEALTH OUTLOOK** for patients with asthma has become better, become worse, or has stayed about the same?

1 Become better **GO TO Q6A, THEN Q7**
2 Become worse **SKIP TO Q6B**
3 About the same **SKIP TO Q7**
8 (VOL) Don't know **SKIP TO Q7**
9 (VOL) Refused **SKIP TO Q7**

*Questions 2 and 4 were intentionally removed from the questionnaire during finalisation by the GAPS team.

ASK IF Q5=1:

Q6a. Why has the long-term health outlook improved for patients with asthma?
[DO NOT READ LIST. MULTIPLE RECORD]

1 Better medications for asthma
2 More asthma medications
5 Better treatment of comorbidities associated with asthma
6 More specialists for asthma patients
7 Better understanding of the disease by physicians
12 Better understanding of the disease by patients
13 Better engagement of patients in managing their disease
14 Better patient adherence with treatment regimens
15 Less smoking or exposure to passive smoking
16 Less pollution
17 More control of triggers or environmental allergens
97 Other (SPECIFY) **Verbatim variable: Q6a_OTH**
98 (VOL) Don’t know
99 (VOL) Refused

SKIP TO Q7

ASK IF Q5=2:

Q6b. Why has the long-term health outlook worsened for patients with asthma?
[DO NOT READ LIST. MULTIPLE RECORD]

1 Worse medications for asthma
2 Fewer asthma medications
5 Worse treatment of comorbidities associated with asthma
6 Fewer specialists for asthma patients
7 Worse understanding of the disease by physicians
12 Worse understanding of the disease by patients
13 Less engagement of patients in managing their disease
14 Worse patient adherence with treatment regimens
15 More smoking or exposure to passive smoking
16 More pollution
17 Less control of triggers or environmental allergens
97 Other (SPECIFY)
98 (VOL) Don’t know
99 (VOL) Refused

Q7. **[IF COUNTRY is not China**: For the next question, by maintenance medication, we mean inhaled corticosteroids, leukotrienes, or inhaled corticosteroid/long-acting beta agonist (ICS/LABA) combinations.

[IF COUNTRY is China: For the next question, by maintenance medication, we mean oral corticosteroids, inhaled corticosteroids, leukotrienes, long-acting anti-cholinergics, oral beta agonists or inhaled corticosteroid/long-acting beta agonist (ICS/LABA) combinations.]

Approximately what percent of your adult asthma patients are on maintenance medication for their asthma?

[INTERVIEWER INSTRUCTION: Confirm after data entry].

Percent: ________ [RANGE: 0-100]

998 (VOL) Don't know
999 (VOL) Refused

Q8. In your experience, thinking about your adult asthma patients overall, how much of a problem are the following when it comes to patient adherence with their medication regimens for asthma? **[READ ITEM]** Is this a major problem, a minor problem, or not a problem to patients' adherence?

RANDOMIZE ORDER

a. Troublesome side effects
b. Patients don't perceive the benefit of the treatment
c. The cost of medications
d. Inconvenience of dosing schedule
e. Low patient education level or poor understanding of disease
f. Poor inhaler technique
g. Forgetfulness
h. Low level of patient engagement in disease
i. Corticosteriods are perceived as harmful
j. Patients only take treatment when needed
k. Patients believe asthma symptoms are normal

1 Major problem
2 Minor problem
3 Not a problem
8 (VOL) Don’t know
9 (VOL) Refused

READ: The next questions ask about asthma management practices. Since this study is being conducted in multiple countries and in a variety of clinical settings, these approaches may or may not be relevant to your healthcare setting.

Q9. For what percent of your adult asthma patients do you implement a written asthma action plan?

[**INTERVIEWER INSTRUCTION**: Confirm after data entry].

Percent: ________ [RANGE: 0-100]

998 (VOL) Don't know
999 (VOL) Refused

Q10a. **[IF COUNTRY is not China**: For the next question, by maintenance medication, we mean inhaled corticosteroids, leukotrienes, or inhaled corticosteroid/long-acting beta agonist (ICS/LABA) combinations.

[IF COUNTRY is China: For the next question, by maintenance medication, we mean oral corticosteroids, inhaled corticosteroids, leukotrienes, long-acting anti-cholinergics, oral beta agonists or inhaled corticosteroid/long-acting beta agonist (ICS/LABA) combinations.]

Do you ever estimate your patients' adherence with their MAINTENANCE treatments?

1	Yes	
2	No	**SKIP TO Q11a**
8	Don't know	**SKIP TO Q11a**
9	Refused	**SKIP TO Q11a**

Q10b. How do you estimate your patients' adherence with MAINTENANCE treatments? Do you…? **[READ LIST; MULTIPLE RECORD**]

1 Estimate based on patient interview
2 Ask the patient directly
3 Examine prescription labels for date dispensed
4 Check pharmacy records
5 Check with family member
6 Estimate based on the timing of the request for refill
97 OR use some other method (SPECIFY)
98 (VOL) Don't know
99 (VOL) Refused

Q11a. Do you ever estimate your patients' use of RELIEVER treatments?

1	Yes	
2	No	**SKIP TO Q12a**
8	(VOL) Don't know	**SKIP TO Q12a**
9	(VOL) Refused	**SKIP TO Q12a**

Q11b. How do you estimate your patients' use of RELIEVER treatments? Do you…? [**READ LIST; MULTIPLE RECORD**]

1 Estimate based on patient interview
2 Ask the patient directly
3 Examine prescription labels for date dispensed
4 Check pharmacy records
5 Check with family member
97 OR use some other method (SPECIFY)
98 (VOL) Don't know
99 (VOL) Refused

Q12a. How do you assess asthma control in your adult asthma patients?
[DO NOT READ LIST. MULTIPLE RECORD]

1 Frequency of symptoms
2 Frequency of nighttime awakenings
3 Interference with normal activities (excluding work)
4 Interference with work or household work
5 Lung function with spirometry (FEV1) or peak flow
6 Exacerbations
7 Validated patient-reported outcomes via questionnaires (e.g., Asthma Control Test (ACT), Asthma Control Questionnaire (ACQ))
8 Induced sputum eosinophil measurement
9 Use of reliever SABAs (short acting beta agonist) for symptom control
97 Other (SPECIFY)
98 (VOL) Don't know
99 (VOL) Refused

Q13. Do you use technology to manage your adult asthma patients? By technology we mean mobile applications, web portals or other mobile, online, or digital tools.

1 Yes
2 No
8 (VOL) Don't know
9 (VOL) Refused

Q14a. How often do your adult asthma patients get the treatment that you believe is best for them?

1	Always	**SKIP TO Q15**
2	Most of the time	
3	Some of the time	
4	Never	
8	(VOL) Don't know	**SKIP TO Q15**
9	(VOL) Refused	**SKIP TO Q15**

Q14b. Why do your adult asthma patients not get the treatment that you believe is best for them?
[DO NOT READ LIST. MULTIPLE RECORD]
1 Local guidelines do not recommend
2 Not on hospital/clinic formulary
3 Not on insurance formulary
4 Too expensive for patient
5 Patient prefers other treatment
6 Patient does not understand why it is important to use this medication
97 Other (SPECIFY)
98 (VOL) Don't know
99 (VOL) Refused

SMART DOSING

Q15. Some inhaled corticosteroids/long-acting beta-agonists fixed dose combinations for treating asthma, known as ICS/LABAs, can be used as a reliever PRN medicine in addition to the maintenance usage. This approach is called SMART or MART dosing, which means "Single Maintenance And Reliever Therapy" dosing. Are you aware of this dosing approach for asthma?

1	Yes	
2	No	**SKIP TO Q26a**
8	(VOL) Don't know	**SKIP TO Q26a**
9	(VOL) Refused	**SKIP TO Q26a**

Q16. Do you know which treatments are indicated for this dosing approach for asthma?

[DO NOT READ LIST. MULTIPLE RECORD]
[PROGRAMMING: LABEL EACH TREATMENT ACCORDING TO COUNTRY SPECIFICATIONS values are 0=no, 1=yes, 98=DK, 99=REF]

AUSTRALIA
Symbicort
Seretide
Flutiform
Breo Ellipta

CANADA (Q16CAN1, Q16CAN2…)
Advair
Symbicort
Zenhale

CHINA (Q16CHN1, Q16CHN2…)
Advair
Adoair
Fostair
Foster
Inuvair
Seretide
Symbicort
Viani

FRANCE (Q16FRA1, Q16FR2…)
Atmadisc
Flutiform
Innovair
Seretide
Symbicort

GERMANY (Q16GER1, Q16GER2…)
Atmadisc
Flutiform
Foster
Inuvair
Relvar
Seretide
Symbicort
Viani

97 Other (Specify)
98 (VOL) Don't Know
99 (VOL) Refused

ASK IF Q15=1
Q17. And have you ever prescribed this dosing approach for asthma, that is, using ICS/LABAs as both maintenance and reliever therapy?

1 Yes
2 No **SKIP TO Q26a**
8 (VOL) Don't know **SKIP TO Q26a**
9 (VOL) Refused **SKIP TO Q26a**

Q17b. How have you learned about this dosing approach?
[DO NOT READ; MULTIPLE RECORD]

1 GINA Guidelines
2 Local Guidelines
3 Scientific conferences
4 Pharmaceutical sales representatives
5 Published literature
7 Other (SPECIFY)
8 (VOL) Don't know
9 (VOL) Refused

Q18. When prescribing this dosing approach for asthma, which factors do you consider to be relevant? Again, the dosing approach is using ICS/LABAs as both maintenance and reliever therapy for asthma.
[DO NOT READ LIST; MULTIPLE RECORD]

1 Patients with more mild symptoms (daytime or nighttime symptoms 1 day a week)
2 Patients with moderate symptoms (daytime or nighttime symptoms a 2-3 days a week)
3 Patients with severe symptoms (daytime or nighttime symptoms 4 or more

days a week)
4 Saving the patient money because they only need 1 inhaler
5 More convenient for the patient because they only need 1 inhaler
6 Patients with lots of comorbidities
7 Patients with poor adherence
8 Patients with good adherence
9 Patients who exacerbate or patients at risk of exacerbations
10 Elderly adults
11 Patients less than 18 years of age
97 Some other factor (SPECIFY)
98 (VOL) Don't know
99 (VOL) Refused

Q19. Again, thinking about the dosing approach of using ICS/LABAs as both maintenance and reliever therapy, are there situations in which you would NOT prescribe this dosing strategy for asthma?

1	Yes	
2	No	**SKIP TO Q22**
8	(VOL) Don't know	**SKIP TO Q22**
9	(VOL) Refused	**SKIP TO Q22**

Q20. And what are these situations in which you would NOT prescribe this dosing strategy for asthma?
[DO NOT READ LIST. MULTIPLE RECORD]

1 Patients with more mild symptoms (daytime or nighttime symptoms 1 day a week)
2 Patients with moderate symptoms (daytime or nighttime symptoms a 2-3 days week)
3 Patients with severe symptoms (daytime or nighttime symptoms 4 or more days a week)
4 Saving the patient money because they only need 1 inhaler
5 More convenient for the patient because they only need 1 inhaler
6 Patients with lots of comorbidities
7 Patients with poor adherence
8 Patients with good adherence
9 Patients who exacerbate or are at risk of exacerbations
10 Elderly adults
11 Patients less than 18 years of age
97 Some other situation (SPECIFY)
98 (VOL) Don't know
99 (VOL) Refused

Q21. Approximately what percent of your ICS/LABA fixed dose combination inhaler prescriptions for asthma are prescribed for this dosing approach? Again, the dosing approach is using ICS/LABAs as both maintenance and reliever therapy for asthma.

Percent: ________ [RANGE: 1-100]

998 (VOL) Don't know
999 (VOL) Refused

Q22. When you prescribe ICS/LABA as both maintenance and reliever therapy for asthma, how often do you also prescribe a short-acting beta agonist or a short-acting bronchodilator as a reliever?

1	Always	
2	Most of the time	
3	Some of the time	
4	Never	**SKIP TO Q24**
8	(VOL) Don’t know	**SKIP TO Q24**
9	(VOL) Refused	**SKIP TO Q24**

Q23. And what factors do you consider to be relevant? [That is, when you prescribe an additional reliever OR an additional short-acting bronchodilator as a reliever for asthma when also prescribing ICS/LABA as both maintenance and reliever therapy for asthma.]
[DO NOT READ; MULTIPLE RECORD]

1 Patient familiarity with short-acting-beta medicine
2 Patients want an extra reliever to feel safer
3 Patients are accustomed to having an extra reliever
4 Patient convenience—having extra reliever in car, at work, for travel, etc.
97 Other (SPECIFY)
98 (VOL) Don’t know
99 (VOL) Refused

Q24. I am going to ask again about the dosing approach of using ICS/LABAs as both maintenance and reliever therapy for asthma. In these cases, what dosing instructions do you give to the pharmacy?
[READ LIST; SINGLE RECORD]

1 I don’t write anything different on the prescription than the normal maintenance dosing regimen per label
2 I write Maintenance Plus Reliever language as a standard choice in my prescribing
3 I write ICS/LABA as both maintenance and reliever

4 I have to create my own text noting maintenance frequency and use as needed/when necessary PRN
5 I cannot modify the pre-specified prescription instructions (e.g., electronic prescribing system)
97 Other (SPECIFY) [**PROGRAMMING: ALLOW OTHER TEXT RESPONSE IF RESPONSE 1-5 IS SELECTED]**
[INTERVIEWER: Please capture additional information with Other field)
98 (VOL) Don't know
99 (VOL) Refused

Q25a1. How do you communicate the dosing instructions for asthma to your patients? **[READ LIST; MULTIPLE RESPONSE]**

1 VERBAL instructions to use the ICS/LABA as RELIEF as well as maintenance
2 WRITTEN instructions to use the ICS/LABA as RELIEF as well as maintenance
3 VERBAL instructions to use the ICS/LABA as RELIEF but not maintenance
4 WRITTEN instructions to use the ICS/LABA as RELIEF but not maintenance
97 OR Given some other way (SPECIFY)
98 (VOL) Don't know **SKIP TO Q26a**
99 (VOL) Refused **SKIP TO Q26a**

Q25a2. And in your instructions to the patient, do you specify a daily limit for relief usage?

1 Yes
2 No
98 (VOL) Don't know
99 (VOL) Refused

Q25b. Overall, how well do your adult asthma patients understand these dosing instructions?
[READ LIST]
1 Very well
2 Somewhat well
3 Not very well
4 Not well at all
8 (VOL) Don't know
9 (VOL) Refused

Q25c. When you prescribe ICS/LABAs as both maintenance and reliever therapy for adult asthma patients, do you measure adherence with the dosing strategy that you have prescribed?

1	Yes	
2	No	**SKIP TO Q25e**
3	Don't know	**SKIP TO Q25e**
4	Refused	**SKIP TO Q25e**

Q25d. How do you measure this?
[READ LIST. MULTI RECORD]

1 Estimate based on patient interview
2 Ask the patient directly
3 Examine prescription labels for date dispensed
4 Check pharmacy records
5 Check with family member
6 Estimate based on the timing of the request for refill

97 OR use some other method (SPECIFY)
98 (VOL) Don't know
99 (VOL) Refused

Q25e. Comparing the time and effort it takes to prescribe ICS/LABAs as both maintenance and reliever therapy to the time and effort it takes you to prescribe other asthma treatments, would you say the ICS/LABA combination takes… ?
[READ LIST.]

1 Much more time and effort
2 Somewhat more time and effort
3 Somewhat less time and effort
4 Much less time and effort OR
5 Is it about the same?
8 (VOL) Don't know
9 (VOL) Refused

PROGRAMMING INSTRUCTIONS FOR Q26 SERIES: These are single-response questions but need to be country specific (for example, Q26aCHN, Q26bCHN…)

Q26a. Thinking about all the adult asthma patients you treat, overall which of the following ICS/LABA fixed dose combination inhalers do you prescribe most often for asthma?
[READ LIST; SINGLE RESPONSE]

(RANDOMIZE)
AUSTRALIA
Breo Ellipta

Flutiform
Seretide
Symbicort

CANADA
Advair
Symbicort
Zenhale

CHINA
Foster
Seretide
Symbicort

FRANCE
Flutiform
Innovair
Seretide
Symbicort

GERMANY
Atmadisc
Flutiform
Foster
Inuvair
Relvar
Seretide
Symbicort
Viani

JAPAN
Adoair
Flutiform
Relvar
Symbicort

95 (VOL) None of these **[SKIP TO D1]**
96 (VOL) All about equally **[SKIP TO 26c]**
97 (VOL) Other (SPECIFY)
98 (VOL) Don't know **[SKIP TO D1]**
99 (VOL) Refused **[SKIP TO D1]**

Q26b. And what is next most frequent ICS/LABA fixed dose combination inhaler you prescribe for asthma?
[READ LIST; SINGLE RESPONSE]

(RANDOMIZE)
AUSTRALIA

Breo Ellipta
Flutiform
Seretide
Symbicort

CANADA
Advair
Symbicort
Zenhale

CHINA
Foster
Seretide
Symbicort

FRANCE

Flutiform
Innovair
Seretide
Symbicort

GERMANY
Atmadisc
Flutiform
Foster
Inuvair
Relvar
Seretide
Symbicort
Viani

JAPAN
Adoair
Flutiform
Relvar
Symbicort

95 (VOL) None of these
96 (VOL) All about equally
97 (VOL) Other (SPECIFY)
98 (VOL) Don't know
99 (VOL) Refused

[SKIP Q26c IF Q26a=95/98/99. IF Q26a=97, PLEASE READ-IN SPECIFY VERBATIM. WHEN Q26a=96, PLEASE READ IN "ICS/LABA fixed dose combination inhalers"**]**

Q26c. What is THE MOST important factor to you when prescribing [INSERT Q26A TREATMENT ABOVE]? **[SINGLE RECORD]**
[INTERVIEWER: Ask as open-end question. Read list only when necessary (if Don't know or Depends)]

1 Symptom severity
2 Patients with lots of co-morbidities
3 Patients with more severe lung function impairment
4 Patients with poorer asthma control (e.g., lower scores on the Asthma Control Test)
5 Patients with poor adherence
6 Patients who exacerbate or are at risk of exacerbation
7 Patient inhaler preference
8 Price or cost to the patient
9 Flexibility to change the dose (ICS dose titration)
10 Age of the patient OR
97 Some other factor? (SPECIFY)
98 (VOL) Don't know
99 (VOL) Refused

READ: Now a few last questions for demographic purposes.

D1. What is your age?
______ [RANGE: 1-99]
9999 (VOL) Refused

D2. Since medical school, have you taken any continuing medical education (CME) courses, attended any medical congresses, or attended training related to asthma management and treatment?
1 Yes
2 No
8 (VOL) Don't know
9 (VOL) Refused

D3. Is your primary practice located in a central city, a suburb, a small city, a town, or a rural area?
1 Central city
2 Suburb
3 Small city
4 Town
5 Rural
8 (VOL) Don't know
9 (VOL) Refused

D4. RECORD GENDER. Ask if necessary.

1 Male
2 Female
9 (VOL) Refused

D5. Think about the place where you have most of your contact with patients. What type of practice is this? **[READ LIST]**

1 Single specialty practice
2 Multi-specialty practice
3 Hospital or hospital-based clinic
4 Other (specify)
8 (VOL) Don’t know
9 (VOL) Refused

LAST SCREEN: We have finished the interview. This survey was sponsored by GSK. Thank you very much for your contribution to this study.
