# Supplementary material for: Physician perspectives on the burden and management of asthma in six countries: The Global Asthma Physician Survey (GAPS)
Source: BMC Pulm Med. 2017 Nov 23;17:153. doi: 10.1186/s12890-017-0492-5 (PMC5701503; doi:10.1186/s12890-017-0492-5)
Supplement: Supplementary file 2 — Table S1. Reasons given for the perceived improved outlook for asthma patients in the last 10 years. Table S2. Products approved for maintenance and reliever treatment of asthma in the countries studied. (PDF 202 kb) [file 12890_2017_492_MOESM2_ESM.pdf]

Physician Perspectives on the Burden and Management of Asthma in Six Countries: the Global Asthma Physician Survey (GAPS)

Supplementary Information File 1: Tables of additional information

Supplementary Table 1: Reasons given for the perceived improved outlook for asthma patients in the last 10 years

% of physicians giving response	**Total** N=1489	**Canada** N=247	**France** N=207	**Germany** N=265	**Australia** N=264	**China** N=251	**Japan** N=255
Better and more asthma medication/devices	90	89	76	92	93	95	90
Better understanding of the disease by physicians	22	19	27	4	24	45	2
More specialists for asthma patients	21	14	9	10	29	34	15
Better understanding of the disease by patients	16	5	24	7	-	38	-
Better treatment for comorbidities associated with asthma	16	9	10	12	8	33	5
Education / awareness / understanding (unspecified)	13	49	-	5	61	4	11
Better patient adherence with treatment regimens	13	2	5	10	-	35	-
Better engagement of patients in managing their disease	13	1	20	7	-	29	-
Improved healthcare / follow-up care / diagnostic tools	9	22	1	9	22	-	14
Less smoking or exposure to passive smoking	9	5	7	2	1	24	1
Prevention	1	2	-	-	15	-	-
Other responses combined	23	30	20	15	37	18	29

Only answers with a response rate of ≥15% in at least one country have been included. '-' represents values of 0% or <1%.

Respondents were physicians who believed that the outlook for asthma patients has improved in the last 10 years. Multiple responses were allowed.

Supplementary Table 2: Products approved for maintenance and reliever treatment of asthma in the countries studied

Country	Products approved for MART use	ICS/LABA dosage approved, µg [inhalations/day*]	Indicated age of patients
**Canada**[a]	Symbicort® Turbuhaler® (budesonide/formoterol)	100/6; 200/6 [2 inhalations/day]	≥12 years
**France**[b]	Symbicort® Turbuhaler®	100/6; 200/6 [2 inhalations/day]	≥18 years
	DuoResp® (budesonide/formoterol)	200/6 [2 inhalations/day]	≥18 years
	Fostair®/Innovair® (beclomethasone/formoterol)	100/6 [2 inhalations/day]	≥18 years
**Germany**[c]	Symbicort® Turbuhaler®	200/6 [2 inhalations/day]	≥18 years
	DuoResp®	200/6 [2 inhalations/day]	≥18 years
	Foster®/Inuvair®	100/6 [2 inhalations/day]	≥18 years
**Australia**[d]	Symbicort® Rapihaler®	50/3; 100/3 [4 inhalations/day]	≥12 years
	Symbicort® Turbuhaler®:	100/6; 200/6 [2 inhalations/day]	≥12 years
**China**[e]	Symbicort®	200/6; 100/6 [2 inhalations/day]	≥18 years
**Japan**[f]	Symbicort® Turbuhaler®	200/6 [2 inhalations/day]	≥15 years

ICS, inhaled corticosteroid; LABA, long-acting β_2 agonist; MART, maintenance and reliever therapy.

*Doses given are ex-valve (metered). Number of inhalations per day is the minimum recommended for maintenance therapy.

References:
a) Symbicort® Product Monograph, AstraZeneca Canada, http://www.astrazeneca.ca/en/Our-Medicines/en-Products-AZ#refS, accessed May 2016;
b) Symbicort® Turbuhaler – Résumé des caractéristiques du produit (100/6 microgrammes par dose), downloaded from http://base-donnees-publique.medicaments.gouv.fr/extrait.php?specid=67916395, accessed May 2016; Symbicort® Turbuhaler – Résumé des caractéristiques du produit (200/6 microgrammes par dose), downloaded from http://base-donnees-publique.medicaments.gouv.fr/extrait.php?specid=61237448, accessed May 2016; Duoresp® – Résumé des caractéristiques du produit, downloaded from http://base-donnees-publique.medicaments.gouv.fr/extrait.php?specid=67827270, accessed May 2016; Innovair® Mentions Legales Completes, downloaded from https://www.chiesi.fr/innovair-1006-µgdose, accessed May 2016;
c) Symbicort® Turbuhaler – Summary of product characteristics, downloaded from http://www.fachinfo.de/pdf/006746, accessed May 2016; Duoresp® Spiromax® – Summary of product characteristics, downloaded from http://www.teva.de/produkte-des-pharmaunternehmens-teva-von-a-z/praeparate-von-a z/praeparate/praeparatedaten/detail/backpid-11054/buchstabe-d/pzn-10311037.html, accessed May 2016; Foster® – Summary of product characteristics, September 2014, https://www.diagnosia.com/de/medikament/foster-1006-mikrogramm-druckgasinhalation, accessed May 2016;

d) Symbicort Rapihaler® Product Information, AstraZeneca Australia, downloaded from http://www.astrazeneca.com.au/home/SearchResult?keyword=symbicort&form-to-process=SearchForm&submit=Go, accessed May 2016; Symbicort Turbuhaler® Product Information, downloaded from http://www.astrazeneca.com.au/home/SearchResult?keyword=symbicort&form-to-process=SearchForm&submit=Go, accessed May 2016;
e) http://www.mims.com.cn/china/drug/info/symbicort%20turbuhaler/?type=full#Indications, accessed May 2016);
f) Symbicort Turbuhaler® Product Information, Japan.
